# Cervical Dystonia with Classic Sensory Tricks and Forcible Sensory Trick Showed Different Functional Connectivity Alterations: A Functional Near-Infrared Spectroscopy Study

**DOI:** 10.3390/jcm15124735

**Published:** 2026-06-18

**Authors:** Xiaofeng Huang, Min Wang, Da Wang, Tao Li, Zhanhua Liang

**Affiliations:** Department of Neurology, First Affiliated Hospital of Dalian Medical University, Dalian 116011, China; huangxiaofeng@firsthosp-dmu.com (X.H.); wangmin7723@renji.com (M.W.); wangda_clair@163.com (D.W.)

**Keywords:** cervical dystonia, sensory trick, functional near-infrared spectroscopy, resting-state brain functional connectivity, sensorimotor integration

## Abstract

**Background/Objectives**: Brain dysfunction and symptoms can be improved with a sensory trick (ST) in more than 80% of patients with cervical dystonia (CD). This study aimed to investigate the functional connectivity (FC) of CD patients with different types of STs using functional near-infrared spectroscopy (fNIRS) and to explore the underlying neural mechanisms of STs. **Methods**: In this study, 35 CD patients (including 15 with classic STs, 15 with forcible STs, 5 with non-STs) and 29 healthy controls (HCs) underwent resting-state fNIRS. We subsequently analyzed FC differences between the groups and their correlations with clinical characteristics. **Results**: The grand-average FC was significantly higher in the non-ST group than in the forcible ST group. Furthermore, compared to the ST group, the non-ST group exhibited significantly increased FC, primarily involving the prefrontal and sensorimotor networks. In the forcible ST group, this hypoconnectivity was negatively correlated with disease severity scores. **Conclusions**: This study supports the concept of CD as a networkopathy, suggesting that both the severity and topology of cortical coherence impairment are modulated by the ST phenotype.

## 1. Introduction

Sensory tricks (STs), also known as alleviating maneuvers, refer to postures or movements that improve dystonia. STs occur in more than 80% of patients with idiopathic cervical dystonia (CD) [1,2] and are not only a hallmark symptom but also a widely utilized clinical marker for the diagnosis and differential diagnosis of CD. Patients can voluntarily relieve dystonia by performing STs. According to the method of application, STs are classified into classic STs and forcible STs [3,4,5]. The former alleviates abnormal head deviations through light touch, whereas the latter requires additional force [5]. Classic STs account for approximately 42.1–58% of cases, while forcible STs account for approximately 42–48.1% [2,4,5,6].

The underlying mechanisms of STs are still poorly understood. Current evidence suggests that efficacy of STs involves the sensorimotor and parietal cortices [7,8]. Specifically, Ramos et al. proposed that ST-induced modulation of the premotor cortex (PMC) during sensorimotor integration shifts the facilitation to inhibition balance towards a “normalized” state [9]. While one study posited that STs are initiated by a slight reactive force or active contralateral head rotation [10], others argue they are linked to motor readiness rather than sensory processing [11,12]. Furthermore, adding to the complexity, an fMRI study revealed decreased sensorimotor network connectivity specifically in CD patients capable of performing STs compared to those without them [13]. Recently, Lee et al. reported significant differences in corticomuscular synchronization during ST performance between the classic and forcible types [14]. This finding implies that the pathophysiology of different ST phenotypes involves varying degrees of sensorimotor integration and complex dynamic mechanisms. Further research is needed to clarify these processes. However, despite these advances, the precise neural basis of STs remains elusive and contentious. Current evidence points toward intricate interactions between the sensorimotor and premotor networks, but how these regions dynamically regulate integration across distinct ST phenotypes is still poorly understood. Therefore, further investigation utilizing functional connectivity approaches is warranted to elucidate the specific neural mechanisms underlying different ST subtypes.

Conventional neuroimaging modalities typically require strict head immobilization, a challenging demand for patients with CD who experience involuntary head movements. Furthermore, CD symptoms often ameliorate in the supine position [1], a confounding factor that likely contributes to the inconsistent findings of previous studies relying on recumbent scanning. In contrast, functional near-infrared spectroscopy (fNIRS) is remarkably tolerant of motion artifacts and permits assessments in an upright posture. This enhances ecological validity for a task-specific dystonia like CD [15,16]. Given these advantages, resting-state functional connectivity (rs-FC), which reflects intrinsic brain network organization, is particularly crucial for investigating CD [17,18]. Therefore, this study utilized fNIRS to characterize rs-FC across different ST phenotypes in CD patients. Specifically, we evaluated both global FC strength and inter-regional connectivity and assessed their correlations with clinical characteristics (e.g., age of onset). We hypothesized that distinct ST phenotypes exhibit specific alterations in brain network connectivity.

## 2. Materials and Methods

### 2.1. General Information

From November 2022 to June 2023, 35 CD patients from the botulinum toxin (BoNT) Injection Clinic of the First Affiliated Hospital of Dalian Medical University and 29 healthy controls (HCs) were continuously recruited. CD was diagnosed according to the 2020 Chinese expert consensus on the diagnosis of dystonia and categorized into adult-onset, focal, and simple types [19]. When identifying the ST type, patients were granted adequate time to demonstrate their alleviating maneuvers, and detailed clinical assessments of the manner, force, direction and effectiveness of the STs were performed by two experienced specialists to ensure accurate grouping. STs were classified as classic STs if they were effective with a light touch, as forcible STs if they required applied force, and as non-STs if they were ineffective regardless of the maneuver [20]. Disease severity was assessed via the Tsui score and the Toronto Western Spasmodic Torticollis Rating Scale (TWSTRS).

Patients were excluded if they met any of the following criteria: (a) were ≥75 years; (b) had other forms of dystonia beyond CD; (c) had received a BoNT injection or taken any medication that affects brain function within 3 months before fNIRS evaluation; (d) had evidence of traumatic brain injury, dementia, essential tremor, other neurological disorders or psychiatric disorders; (e) had a history of medication use before the onset of CD; (f) had a family history of movement disorders; (g) had symptoms too severe to allow their cooperation with the study; or (h) were left-handed. All the subjects voluntarily participated in the study and signed informed consent forms. The study was carried out in accordance with the latest version of the Declaration of Helsinki and was approved by the Ethics Committee of the First Affiliated Hospital of Dalian Medical University (No. PJ-KS-KY-2022-377).

### 2.2. Image Data Collection

**Figure 1:**
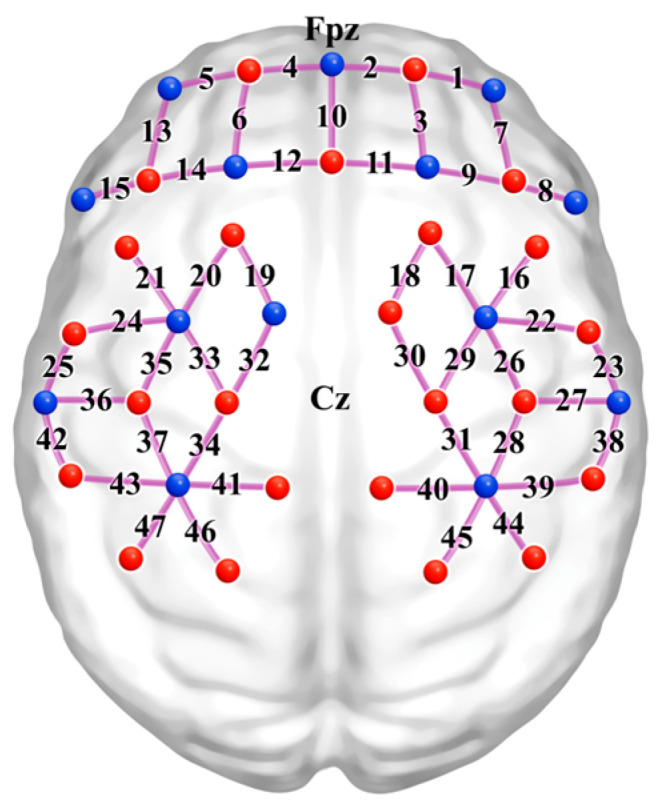
fNIRS channel configuration setup. Sources, detectors, and channels are marked with red circles, blue circles, and purple lines, respectively.

Cortical concentrations of oxyhaemoglobin (HbO) and deoxyhaemoglobin (HbR) were recorded using a portable fNIRS system (NirSmart, Danyang Huichuang Medical Equipment Co., Ltd., Danyang, China) [21]. The device employs near-infrared light-emitting diodes (wavelengths: 730 nm and 850 nm) and avalanche photodiodes detectors, operating at a sampling rate of 11 Hz. Participants wore an fNIRS cap positioned according to the international 10–20 system and cranial anatomy landmarks. The probes array consisted of 23 emitters and 15 detectors (spaced 3 cm apart), forming 47 effective channels (Chs). Spatial alignment was anchored by placing detector D2 at Fpz and the midpoint between emitter S13 and emitter S14 at Cz, primarily covering the frontal and parietal cortices. Probe locations were registered and transformed into Montreal Neurological Institute (MNI) coordinates using NirSpace software (V1.1.4, Danyang, China) [22] (Figure 1). Based on the MNI coordinates, the 47 channels were anatomically assigned to the following regions: orbitofrontal cortex (OFC; Chs 1, 2, 4, 5), frontopolar cortex (FPA; Chs 3, 6,10, 11, 12), dorsolateral prefrontal cortex (DLPFC; Chs 7, 9, 13, 14, 16, 21), triangular part of Broca’s area (Chs 8, 5), frontal eye field (FEF; Chs17, 19, 20), PMC (Chs 18, 22, 24, 26, 29, 30, 32, 33, 35), primary motor cortex (S1; Chs 23, 28, 31, 34), subcentral area (SCA; Chs 25), primary somatosensory cortex (PSC; Chs 27, 36, 37, 39, 40, 41, 42, 43), supramarginal gyrus part of Wernicke’s area (SMG) (Chs 38) and secondary sensory cortex (S2; Chs 44, 45, 46, 47).

The centroid of each channel was defined as the primary brain region detected. To minimize signal occlusion, the optodes were adjusted and participants’ hair was carefully parted. The NirSmart system automatically adjusted source power and detector gain to ensure optimal signal quality. fNIRS data were collected for five minutes in a quiet, lightproof environment. Participants were instructed to relax with their eyes closed and maintain a state devoid of structured thought (clearing the mind). Crucially, they were instructed not to deliberately control or correct their head posture. For patients with cervical dystonia, forcibly trying to hold the head in a midline position requires intense voluntary muscle contraction against the dystonia, which would constitute a motor task and confound the resting-state data. Therefore, patients were allowed to let their head rest naturally in their involuntary dystonic posture. They were explicitly instructed not to voluntarily worsen the deviation or perform any active head movements. The rest of their body was to remain as still as possible.

### 2.3. Image Data Processing

Raw data were imported into NirSpark software (Danyang, China) for analysis [23,24]. The preprocessing pipeline began by converting light intensity into optical density. To mitigate the influence of extracerebral contamination (e.g., scalp blood flow), a long-channel correction was applied. This method estimates the extracerebral signal component derived from channels with large source–detector separations and subtracts it to isolate the cortical signal. This method is a common practice when short separation channels are not available, although it is an approximation and may not completely eliminate extracerebral effects. Subsequently, motion artifacts were identified and corrected using a spline interpolation algorithm for signal segments exceeding six standard deviations [25,26]; this effectively isolates artifacts without distorting the remaining time series. Finally, a bandpass filter (0.01–0.2 Hz) was applied to remove physiological noise, including cardiac (heartbeat), respiratory (breathing), and low-frequency drift components [26]. Hemoglobin concentrations for HbO and HbR were calculated using the modified Beer–Lambert law, with the differential path factor set to 6 for both 730 nm and 850 nm wavelengths. The resulting time series were used to compute functional connectivity between all 47 channels using Pearson’s correlation coefficients. This procedure yielded a 47 × 47 connectivity matrix for each participant. To enable parametric statistical analysis, these correlation coefficients were converted to z-scores using Fisher’s r-to-z transformation [23]. Notably, hemispheric normalization (i.e., “flipping” the left and right hemispheres according to the clinically symptomatic side) was not performed. Given that this study focused on network-level functional connectivity, which typically involves bilaterally symmetric cooperative patterns, rather than localized lateralized activation, group comparisons were conducted using standard anatomical channel coordinates without lateralization adjustment.

### 2.4. Statistical Analysis

Continuous variables following a normal distribution were presented as the means ± standard deviations (SD); otherwise, the median and interquartile range were used. Categorical variables were compared using Fisher’s exact test. For group comparisons of continuous variables, a one-way analysis of variance (ANOVA) with Bonferroni post hoc correction was applied for normally distributed data with homogeneity of variance; Welch’s ANOVA with Dunnett’s T3 post hoc test was used when the assumption of equal variances was violated. For non-normally distributed data, the Kruskal–Wallis test was employed. Bivariate correlations between continuous variables were assessed using Pearson’s correlation coefficient or Spearman’s rank correlation, depending on data distribution. Furthermore, covariance analysis was performed to control for potential confounding of other variables.

Based on the individual z-scored functional connectivity matrices, a group-level FC matrix was generated for each group using NirSpark [23,24]. The FC values of all 47 × 47 pairs (representing 1081 unique connection pairs) were extracted for statistical analysis. First, a one-way ANOVA was performed across the three groups to identify differentially connected pairs. To control for multiple comparisons across the 1081 tests, False Discovery Rate (FDR) correction with the Benjamini–Hochberg procedure was applied, with statistical significance set at *p* < 0.05 (FDR-corrected). Additionally, to explore symptom-specific neural mechanisms, Spearman’s rank correlations were calculated between clinical characteristics and the FC values (z-scores) of all channel pairs specifically within the forcible ST group.

## 3. Results

### 3.1. Participant Characteristics

There were no significant differences among the groups regarding age, sex, disease duration, age at onset, disease severity score, the presence of tremor and head turning (Table 1).

### 3.2. Functional Connectivity

#### 3.2.1. All-Channel Average Functional Connectivity of the Groups

**Figure 2:**
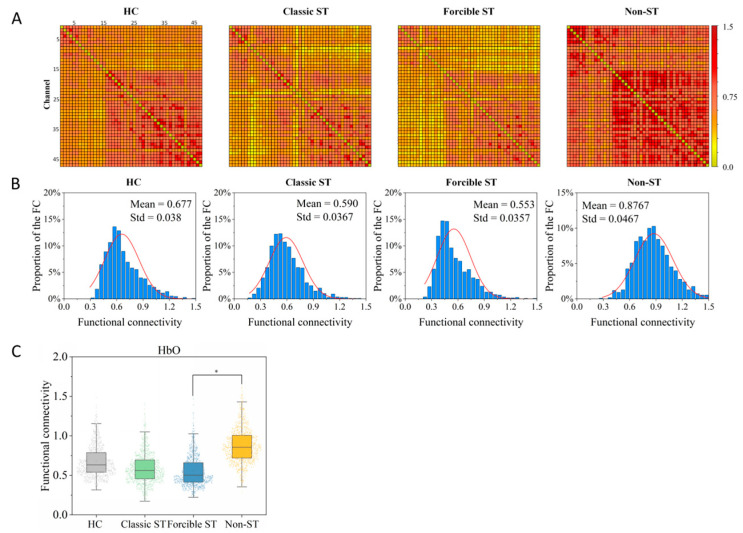
Grand-average functional connectivity (FC) across groups based on oxyhemoglobin (HbO) data. (**A**) Correlation coefficient matrices for each group. The diagonal elements (self-connections) are set to zero. (**B**) Histograms illustrating the FC distribution, plotted as mean ± standard deviation. (**C**) Group differences in global FC values (*: *p* < 0.05).

To compare global FC across groups, we averaged all correlation coefficients in the FC matrices (Figure 2A). The overall FC values ranked from highest to lowest as follows: Non-ST > HC > classic ST > Forcible ST. This pattern was consistent across both the HbO and HbR data. The mean FC values and standard deviations are presented in Figure 2B. One-way ANOVA and Bonferroni post hoc correction were used for group compares, as shown in Figure 2C. Compared with the non-ST group, the forcible ST group presented significantly lower FC values based on HbO data (*p* < 0.05). No significant group differences were observed in the HbR data. We also calculated the grand-average values of all CD patients (data: 0.62 ± 0.26; HbR data: 0.24 ± 0.14).

#### 3.2.2. Channel-Based Functional Connectivity

**Figure 3:**
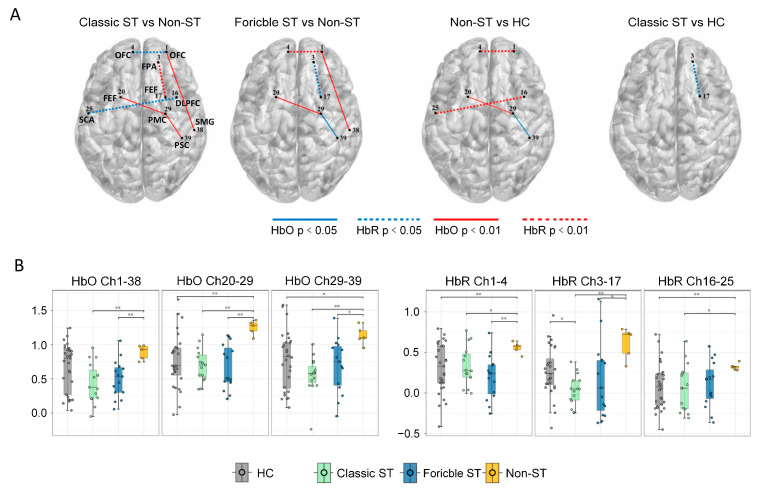
Channel-based functional connectivity. (**A**) Channels with significant differences in FC. Significant connections are indicated by lines: solid lines represent HbO and dashed lines represent HbR; blue indicates *p* < 0.05, and red indicates *p* < 0.01. Numbers in the image represent channels. All *p* values are FDR-corrected. Abbreviations: OFC, orbitofrontal cortex; FPA, frontopolar area; FEF, frontal eye field; DLPFC, dorsolateral prefrontal cortex; PMC, premotor cortex; PSC, primary somatosensory cortex; SMG, supramarginal gyrus; SCA, subcentral area. (**B**) Pairwise differences in FC values (z-scores) across groups. *: *p* < 0.05, **: *p* < 0.005.

Channel-based FC differences among the groups are illustrated in Figure 3A, with the corresponding FC values detailed in Table 2 and Figure 3B. For HbO data, the Non-ST group exhibited significantly greater FC than the classic ST, forcible ST, and HC groups in three channel pairs: Ch20 (FEF)-Ch29 (PMC), Ch29 (PMC)-Ch39 (PSC), and Ch 1(OFC)-Ch 38 (SMG). Similarly, analysis of HbR data revealed that the Non-ST group demonstrated significantly higher FC than the other three groups, for the Ch 1(OFC)- Ch4 (OFC), Ch3 (FPA)-Ch17 (FEF), and Ch 16 (DLPFC)-Ch 25 (SCA) connections. Additionally, compared with the HC group, the classic ST group showed decreased FC between Ch3 (FPA) and Ch17 (FEF).

#### 3.2.3. Relationships Between FC and Clinical Characteristics

**Figure 4:**
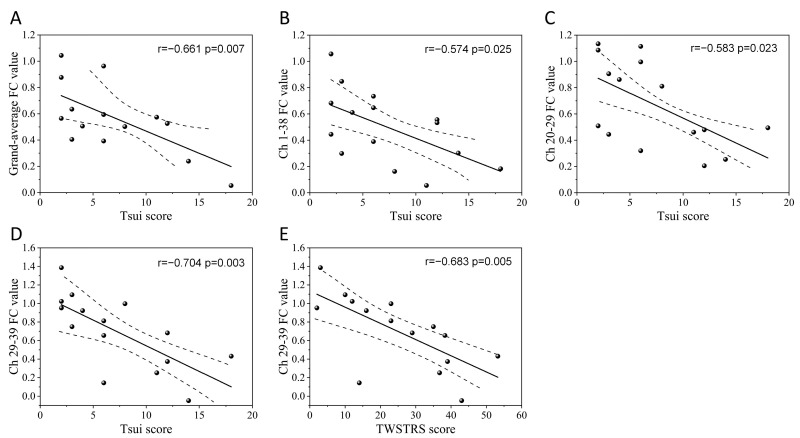
Relationship between FC and Clinical characteristics in the forcible ST group. (**A**) Correlation between global (grand-average) FC and the Tsui score. (**B**) Correlation between Ch1–38 FC (z-score) and the Tsui score. (**C**) Correlation between Ch20–29 FC (z-score) and the Tsui score. (**D**) Correlation between Ch29–39 FC (z-score) and the Tsui score. (**E**) Correlation between Ch29–39 FC (z-score) and the TWSTRS score.

Based on the HbO data, FC values were significantly correlated with disease severity in the forcible ST group. Both the grand-average and FC values of specific abnormal channels showed negative correlation with the Tsui score. Additionally, the FC between Ch 29 and Ch 39 was also negatively related with the TWSTRS score (Figure 4). Based on the HbR data, the FC value of Ch 16 and Ch 25 was positively correlated with the age of onset (*p* = 0.013, r = 0.951); however, this correlation was not significant after FDR correction (*p* > 0.05). The full set of correlations between abnormal FC values and clinical characteristics is presented in Table 3. There was no significant correlation between the age of onset, disease duration, and FC in the three CD groups. Furthermore, the FC of Ch 1, Ch 4, Ch3 and Ch 17 based on HbR did not show a significant correlation with any of the clinical characteristics.

## 4. Discussion

This study revealed decreased overall FC, especially among the PFA, S1 and PMC in CD patients with STs. Conversely, CD patients without effective STs exhibited enhanced FC compared to both healthy controls and CD patients with effective STs which, involving the PMC, PSC, and PFA, may reflect a loss of inhibition at the cortical level within the sensorimotor network. This aligns with prior fMRI evidence demonstrating that, compared with healthy controls, CD patients without STs exhibited increased FC not only within the sensorimotor network (particularly in the premotor and S1) but also between the primary motor cortex and the parietal, temporal, and cerebellum regions. This was observed both during normal resting state and during resting state with STs (where healthy controls and patients without STs mimicked the trick postures) [13]. Furthermore, the same study indicated that patients without an ST exhibited enhanced FC between the motor cortex and parietal, temporal, and cerebellar regions at resting state compared with CD patients with an ST [13]. These findings collectively suggest that a global increased FC in sensorimotor network in CD patients may be driven primarily by the non-ST group, a notion strongly supported by our results. Our previous study demonstrated that in CD patients with forcible STs, the supplementary motor area contralateral to the head turn exhibited significantly greater excitability compared to the ipsilateral side [27]. Conversely, patients with a poor ST efficacy demonstrate impaired short-term discriminatory ability, suggesting deficient proprioceptive processing [28]. The execution of an ST dynamically modulates cortical activity: PET studies indicate that performing an ST increases activation in the ipsilateral parietal cortex while decreasing activation in the contralateral SMA and primary sensorimotor cortex [7]. Furthermore, TMS studies reveal that STs mitigate the hyper-facilitation of the motor cortex [29], and EEG/MEG evidence shows that STs increase inter-cortical coherence, which subsequently reduces corticomuscular coherence and alleviates dystonia [14]. Synthesizing these findings, the neural mechanisms by which STs alleviate the CD symptoms are multifaceted. They encompass the recalibration of the sensorimotor network, the restoration of proprioceptive processing and neuroplasticity, the re-engagement of descending inhibitory pathways, and the modulation of cognitive–motor interactions. These mechanisms interact synergistically to mitigate dystonic symptoms. Future research should aim to disentangle the specific spatiotemporal dynamics and causal interrelationships among these mechanisms.

Importantly, previous studies have largely overlooked the underlying differences in neural mechanisms between classic and forceful STs. In this study, we found that compared with the non-ST group, the forceful ST group exhibited decreased FC within the bilateral OFC, between the OFC and SMG, and within the sensorimotor network; meanwhile, the classic ST group showed decreased FC between the SCA and DLPFC. Although no significant FC differences were observed between the forcible ST and classic ST groups, which may be attributable to limited statistical power, the distinct patterns hint at divergent pathophysiology. This is supported by an EEG-EMG study demonstrating that only the classic ST group exhibited a significant decrease in corticomuscular coherence during ST performance, which suggested that classic and forcible STs may rely on distinct mechanisms at the central (brain) and peripheral (muscle) levels [14]. These findings suggest that the effects of an ST extend beyond simple static mechanical counter-pressure. The precise execution of a sensory trick requires a sequence of motor preparation, execution, and sensory monitoring [30]. In the context of reduced intercortical FC, which inherently limits the coordination between corresponding brain regions, a greater degree of exogenous sensory stimulation may be required to compensate for this sensorimotor network dysfunction. This heightened sensory input likely serves to override deficient intracortical inhibition, thereby reducing abnormal muscle spasms and enabling the execution of the ST.

FC strength was significantly correlated with disease severity. This aligns with findings from task fMRI studies involving hand movements, which revealed that greater CD symptom severity was associated with reduced functional activity in the somatosensory cortex and lower hemodynamic responses within the primary sensorimotor network [31,32]. In the present study, although the Tsui and TWSTRS scores were higher in the forcible ST group than in the classic ST group, the differences were not significant. Furthermore, previous studies have indicated that the age at onset in patients with an ST was lower than those without an ST [4], suggesting that age at onset may impact ST efficacy or brain FC in CD patients. Interestingly, we observed that abnormal hyperconnectivity between the DLPFC and sensorimotor cortex showed a positive correlation with age of onset; however, this correlation was not statistically significant after FDR correction. Further studies with larger cohorts are warranted to elucidate how age of onset influences ST effectiveness and functional network architecture in CD.

A notable finding in our study is the asymmetry between the HbO and HbR results, with connectivity differences being more pronounced and statistically significant in the HbO signal, while the corresponding HbR effects were weaker and often non-significant. Several factors may contribute to this discrepancy. Physiologically, the neurovascular coupling response is typically characterized by a larger and more reliable increase in HbO compared to the smaller and more variable changes in HbR, making HbO a more sensitive marker of neuronal activation. Methodologically, HbR signals have a lower signal-to-noise ratio and are more susceptible to physiological artifacts (e.g., blood pressure and respiration fluctuations), which can obscure underlying neural signals and reduce statistical power. While the weaker HbR results introduce a degree of uncertainty and warrant cautious interpretation, the convergence of evidence from the HbO analysis, combined with the large effect sizes and biological plausibility of our findings, supports the overall conclusion of altered prefrontal–sensorimotor connectivity across different CD subgroups. Future research should aim to replicate these findings using more advanced fNIRS methodologies, such as time-resolved fNIRS or systems with short-separation channels to better eliminate extracerebral contamination, and isolate the specific effects of the sensory trick phenotype while controlling for clinical confounders like anxiety and tremor severity.

Several limitations of this study should be acknowledged. First, the sample size is relatively small, particularly in the non-ST group. The results are exploratory and require confirmation in larger cohorts. Due to this limited sample size, we did not perform multivariate regression to control for potential confounding variables known to influence brain connectivity, such as anxiety, depression, pain, sleep quality, disease subtype, tremor severity, and head movement load. Consequently, the observed group differences should be interpreted with caution, as they may be influenced by these unmeasured factors. Second, our fNIRS system lacked short-separation channels, which are the gold standard for regressing out superficial systemic physiological noise. While we applied a long-channel correction method to estimate and subtract extracerebral contamination, this approximation lacks anatomical specificity and may not completely eliminate all superficial effects. Furthermore, band-pass filtering (0.01–0.2 Hz) does not fully resolve this issue, as systemic fluctuations (e.g., Mayer waves, autonomic vasomotion) spectrally overlap with neurovascular low-frequency oscillations [33]. Given that muscle contractions and stress in CD can trigger autonomic arousal, we cannot definitively exclude the possibility that some observed connectivity differences partially reflect residual superficial dynamics rather than pure cortical connectivity. Third, we lacked simultaneous electromyography (EMG) recordings. In CD, abnormal muscle activation or dystonic spasms can induce local blood flow changes in the scalp and cervical muscles, which may be mistakenly detected as cortical activity. Although we instructed patients to relax and applied strict preprocessing, we were unable to objectively quantify subclinical muscle activity. Thus, we cannot completely dissociate the observed cortical connectivity from potential peripheral muscle contributions. Fourth, fNIRS is inherently unable to measure deep brain structures. The pathophysiology of CD is intimately linked to the basal ganglia, thalamus, and cerebellum. By relying solely on cortical fNIRS signals, our conclusions regarding the neural mechanisms of CD are confined to the cortical level. Future multimodal imaging studies (e.g., combining fNIRS with fMRI) are required to construct a complete model of the dystonia network. Fifth, channel data were not aligned (“flipped”) according to the clinically dominant symptomatic hemisphere. Because our initial analytical strategy focused on global functional connectivity rather than lateralized regional activation, we analyzed standard anatomical coordinates. However, CD is inherently lateralized; by averaging channels without aligning to the symptomatic side, we may have mixed distinct connectional fingerprints (e.g., dystonic vs. compensatory hemispheres), thereby increasing inter-subject variance and potentially diluting lateralized connectivity differences. Future studies should employ hemispheric normalization or stratify patients by laterality. Sixth, our clinical characterization lacked detailed stratification by specific movement phenotypes (e.g., tonic, phasic, tremor-dominant). Grouping these phenotypes together may have obscured subtle connectivity changes unique to each subtype. Additionally, the classification of sensory trick subtypes, particularly the ‘forced’ variant, relied on clinical observation rather than objective quantification (e.g., force sensors), making it susceptible to observer bias. Finally, the cross-sectional design limits causal inferences. While we identified distinct functional connectivity patterns associated with different sensory trick phenotypes, we cannot determine whether these neural differences cause specific sensory trick behavior or are a consequence of long-term adaptation. Future longitudinal or interventional studies are warranted to establish these causal mechanisms. This study investigated the potential mechanisms of ST based on resting-state FC, but it cannot reflect the synchronous changes in brain networks during the execution of ST. Therefore, future studies with larger samples and more sophisticated paradigms are needed to directly investigate the active mechanisms during the execution of sensory tricks.

## 5. Conclusions

In this study, fNIRS was utilized to investigate rs-FC alterations in CD patients across different sensory trick phenotypes. By exploring network attributes, we observed significant between-group differences, indicating phenotype-specific alterations in cortical functional connectivity. While these findings provide promising initial insights into the neural mechanisms underlying distinct STs, they must be interpreted with caution. Future studies with larger cohorts, standardized protocols, and external validation are essential to confirm these preliminary results and to evaluate the potential of fNIRS as a clinical tool for patient stratification.

## Figures and Tables

**Table 1:** Demographic and clinical characteristics of the participants.

Variables	HC	CD			*p*-Value
	N = 29	N = 35			
		Classic ST	Forcible ST	Non-ST	
		N = 15	N = 15	N = 5	
Age	49.76 ± 16.34	53.07 ± 11.73	53.93 ± 10.15	50.20 ± 13.07	0.76 ^1^
Sex M/F	11/18	3/12	5/10	2/3	0.71 ^2^
Age of onset	-	45 [30, 53]	46 [43, 49]	40 [31.5, 53]	0.86 ^3^
Disease duration	-	10 [2, 8]	10 [2, 12]	8 [6, 11]	0.95 ^3^
Tsui	-	5.00 ± 3.00	7.27 ± 5.04	5.00 ± 2.83	0.27 ^1^
TWSTRS	-	14 [8, 30.75]	23 [12, 38.25]	21.5 [8.5, 29.75]	0.42 ^3^
Tremor (yes/no)	-	11/4	8/7	3/2	0.52 ^2^
Head-turning (L/R)	-	6/4	5/6	2/2	0.87 ^2^

Abbreviations: CD: cervical dystonia; HC: healthy controls; ST: sensory trick; Non-ST: patients without ST; TWSTRS: Toronto Western Spasmodic Torticollis Rating Scale; M/F: male/female; L/R: Left/Right; -: not Applicable. ^1^ *p* value from one-way ANOVA. ^2^ *p* value from Fisher’s exact test. ^3^ *p* value from the Kruskal–Wallis test.

**Table 2:** Channel pairs with significantly different FC among four groups.

	Ch.	Groups Comparison	FC Value	*p* (FDR Corrected)	Cohen’s d	95% CI for d
HbO	1–38	Non-ST > classic ST	0.89 ± 0.11 vs. 0.44 ± 0.29	0.001	1.72	0.58, 2.87
		Non-ST > forcible ST	0.89 ± 0.11 vs. 0.50 ± 0.28	0.002	1.55	0.42, 2.67
	20–29	Non-ST > classic ST	1.25 ± 0.11 vs. 0.70 ± 0.24	<0.001	2.52	1.25, 3.8
		Non-ST > forcible ST	1.25 ± 0.11 vs. 0.67 ± 0.33	<0.001	1.96	0.78, 3.14
		Non-ST > HC	1.25 ± 0.11 vs. 0.77 ± 0.40	<0.001	1.35	0.25, 2.44
	29–39	Non-ST > classic ST	1.13 ± 0.14 vs. 0.55 ± 0.28	<0.001	2.269	1.04, 3.50
		Non-ST > forcible ST	1.13 ± 0.14 vs. 0.70 ± 0.40	0.011	0.802	0.12, 2.28
		Non-ST > HC	1.13 ± 0.14 vs. 0.78 ± 0.42	0.012	0.89	−0.09, 1.86
HbR	1–4	Non-ST > classic ST	0.56 ± 0.71 vs. 0.33 ± 0.24	0.026	0.59	−0.45, 1.61
		Non-ST > forcible ST	0.56 ± 0.71 vs. 0.20 ± 0.27	0.001	0.88	−0.17, 1.92
		Non-ST > HC	0.56 ± 0.71 vs. 0.30 ± 0.31	0.004	0.68	−0.29, 1.64
	3–17	Non-ST > classic ST	0.61 ± 0.20 vs. 0.04 ± 0.19	0.004	2.97	1.60, 4.33
		Non-ST > forcible ST	0.61 ± 0.20 vs. 0.16 ± 0.46	0.039	1.08	0.01, 2.15
		HC > classic ST	0.27 ± 0.30 vs. 0.04 ± 0.19	0.022	0.86	0.21, 1.51
	16–25	Non-ST > classic ST	0.33 ± 0.04 vs. 0.07 ± 0.29	0.026	1.01	−0.05, 2.07
		Non-ST > HC	0.33 ± 0.04 vs. 0.08 ± 0.18	0.001	1.48	0.47, 2.49

**Table 3:** Correlations between abnormal FC and clinical characteristics in specific groups.

		HbO				HbR
		Average	Ch. 1–38	Ch. 20–29	Ch. 29–39	Ch. 16–25
Classic ST	Tsui score	-	-	-	-	r = 0.658
		-	-	-	-	*p* = 0.008
		-	-	-	-	(*p*′ = 0.064)
						95% CI [0.22, 0.875]
Forcible ST	Age of onset	-	-	-	-	-
	Disease duration	-	-	-	-	-
	Tsui score	r = −0.661	r = −0.574	r = −0.583	r = −0.704	-
		*p* = 0.007	*p* = 0.025	*p* = 0.023	*p* = 0.003	-
		(*p*′ = 0.028 *)	(*p*′ = 0.05)	(*p*′ = 0.05)	(*p*′ = 0.024 *)	-
		95% CI [−0.876, −0.225]	95% CI [−0.839, −0.088]	95% CI [−0.843, −0.101]	95% CI [−0.893, −0.300]	
	TWSTRS score	r = −0.545	r = −0.606	r = −0.569	r = −0.683	-
		*p* = 0.036	*p* = 0.017	*p* = 0.027	*p* = 0.005	-
		(*p*′ = 0.072)	(*p*′ = 0.068)	(*p*′ = 0.072)	(*p*′ = 0.04 *)	-
		95% CI [0.046, 0.826]	95% CI [−0.853, −0.136]	95% CI [0.081, 0.837]	95% CI [0.263, 0.885]	
Non-ST	Age of onset	-	-	-	-	r = 0.951
		-	-	-	-	*p* = 0.013
		-	-	-	-	(*p*′ = 0.104)
						95% CI [0.856, 0.983]

*p*′: FDR-corrected *p*. * *p* < 0.05.

## Data Availability

The data that support the findings of this study are available on request from the corresponding authors.

## Abbreviations

The following abbreviations are used in this manuscript:

CD	Cervical dystonia
fNIRS	Functional near-infrared spectroscopy
FC	Functional connectivity
ST	Sensory trick
HCs	Healthy controls
PMC	premotor cortex
rs-FC	Functional connectivity in resting state
TWSTRS	Toronto Western Spasmodic Torticollis Rating Scale
HbO	Oxyhaemoglobin
HbR	Deoxyhaemoglobin
Chs	Channels
MNI	Montreal Neurological Institute
OFC	Orbitofrontal cortex
FPA	Frontopolar cortex
DLPFC	Dorsolateral prefrontal cortex
FEF	Frontal eye field
SCA	Subcentral area
PSC	Primary somatosensory cortex
SMG	Supramarginal gyrus part of Wernicke’s area
ANOVA	One-way analysis of variance
FDR	False discovery rate
